# Controversies in the Mechanism of Total Parenteral Nutrition Induced Pathology

**DOI:** 10.3390/children2030358

**Published:** 2015-07-31

**Authors:** Jain Ajay Kumar, Jeffery H. Teckman

**Affiliations:** 1 Department of Pediatrics, St. Louis University School of Medicine, Cardinal Glennon Children’s Medical Center, SSM Cardinal Glennon Hospital 1465 South Grand Blvd., St. Louis, MO 63104, USA; E-Mail: teckmanj@slu.edu; 2 Department of Biochemistry and Molecular Biology, Saint Louis University School of Medicine

**Keywords:** liver, gut, neonatal, parenteral nutrition

## Abstract

Over 30,000 patients are permanently dependent on Total Parenteral Nutrition (TPN) for survival with several folds higher requiring TPN for a prolonged duration. Unfortunately, it can cause potentially fatal complications. TPN infusion results in impairment of gut mucosal integrity, enhanced inflammation, increased cytokine expression and trans-mucosal bacterial permeation. It also causes endotoxin associated down regulation of bile acid transporters and Parenteral Nutrition Associated Liver Disease (PNALD), which includes steatosis, disrupted glucose metabolism, disrupted lipid metabolism, cholestasis and liver failure. Despite multiple theories, its etiology and pathophysiology remains elusive and is likely multifactorial. An important cause for TPN related pathologies appears to be a disruption in the normal enterohepatic circulation due to a lack of feeding during such therapy. This is further validated by the fact that in clinical settings, once cholestasis sets in, its reversal occurs when a patient is receiving a major portion of calories enterally. There are several other postulated mechanisms including gut bacterial permeation predisposing to endotoxin associated down regulation of bile acid transporters. An additional potential mechanism includes toxicity of the TPN solution itself, such as lipid mediated hepatic toxicity. Prematurity, leading to a poor development of bile acid regulating nuclear receptors and transporters has also been implicated as a causative factor. This review presents the current controversies and research into mechanisms of TPN associated injury.

## 1. Introduction

Although use of Total Parenteral Nutrition (TPN) is lifesaving, it is responsible for significant complications resulting in both morbidity and mortality [1,2,3]. TPN administration is known to cause the well-characterized Parenteral Nutrition Associated Liver Disease (PNALD), which includes liver steatosis, inflammation, fibrosis, cholestasis, associated glucose intolerance and dyslipidemia. Animal studies have shown that TPN administration is also associated with significant gut mucosal atrophy [4,5,6,7]. Unfortunately, there are no established ameliorative strategies for TPN associated pathology.

The mechanisms of TPN related injury are likely multifactorial [8] and remain a major research focus in gastroenterology and hepatology. This manuscript seeks to review the current evidence as it relates to the postulated mechanisms involved with TPN related pathology.

## 2. Bile Acid Mediated Farnesoid X Receptor (FXR) Induction of FGF19

In clinical settings, TPN related injury does not develop if enteral nutrition is provided. In fact, it is well established that once cholestasis sets in, its reversal occurs when a patient is receiving all or a majority of calories via the enteral route. A study reviewed 172 neonates on TPN and noted significant differences in the development of cholestasis based on the day enteral feeding was started [9].

Recent insights from cell culture and animal models show that enteral bile acids activate a nuclear receptor, Farnesoid X Receptor (FXR) in intestinal epithelial cells [10,11]. Such activation stimulates production of a growth factor, Fibroblast Growth Factor-19 (FGF19) and its delivery via the portal circulation to the liver [12,13]. FGF19 functions as a secretory signal to the liver, regulating bile acid synthesis via repression of CYP7A1 (Cholesterol 7 alpha-hydroxylase—rate limiting step) [14]. Additionally, in obese mice, intravenous FGF19 prevented or reversed diabetes, improved glycemic control, improved lipid control, reduced hepatic triglyceride levels and reduced hepatic steatosis [15,16,17]. It is thus predicted that hepatic bile acid synthesis, lipid and glucose metabolism is modulated via intestinal FXR signaling [18,19,20]. We have previously published significantly reduced FGF19 levels with TPN use. When animals on TPN were treated with an FXR agonist (Chenodeoxycholic Acid), there was elevation in FGF19 level [21].

Thus, based on current literature, it seems possible that PNALD result from an altered FXR-FGF19 signaling. It is particularly important to note that while, Ursodeoxycholic acid (UDCA) has been used in patients with PNALD with inconsistent results [22,23,24], unlike CDCA, UDCA has minimal activity for FXR [10,20].

## 3. TGR5 and Glucagon Like Peptides

Animal studies indicate that intestinal mucosal atrophy occurs upon TPN infusion, however the mechanisms remain unknown. Rodent studies show that feeding bile acids induces mucosal proliferation [6,25]. A rather interesting result from our recent publication indicates a robust gut growth and near normalization of such atrophy upon treatment with bile acid receptor agonists [21]. It has also been reported that there is a significantly enhanced expression of Glucagon LikePeptides (GLPs) with bile acid treatment in animal models [21]. Data suggests that the Glucagon Like Peptide-1 (GLP-1) regulates insulin, glucose homeostasis and hepatic steatosis [26]. GLP-1 is increasingly recognized as one of the key gut hormones responsible for enhancing the insulin response to nutrient ingestion a phenomenon known as the ‘incretin effect’ [27]. Glucagon LikePeptide-2 (GLP-2) is one of the most important, well-established, gut-trophic factors [25].

GLP-1 and GLP-2 secretion by enteroendocrine cells is under regulation of the bile acid activated G protein-coupled receptor TGR5 [28,29]. TGR5 is highly localized in crypts and is known to modulate gut trophic effects [30,31]. This leads to a thought provoking idea that a disrupted TGR5 signaling and thus decreased GLP levels during TPN administration could additionally contribute to TPN injury.

## 4. Gut Microbiota, Inflammation and FXR, TGR5

Hepatic steatosis, inflammation, fibrosis, glucose intolerance and dyslipidemia are noted with TPN liver injury [7,32]. Intriguingly these phenotypes overlap with Nonalcoholic steatohepatitis (NASH) [33,34].

New data from human and animal studies point to an increasing evidence of a cross talk between the gut and the liver; with modulation of disease pathology by the gut microbiota [35,36]. Though an average person has only a small percentage of body weight attributable to bacteria (approximately two to five pounds of live bacteria) [37], in real cell numbers we are about 90% bacterial and 10% human. [38] Additionally, the microbial genome exceeds the human genome by two orders of magnitude making us genetically 99% bacterial and 1% human [38,39].

Indeed, some recent studies have shown significant changes in gut microbiota with evolving liver cirrhosis. An increased dysbiosis in the form of greater abundance of bacterial colonies of gram negative organisms like *Bacteroidaceae* and *Enterobacteriaceae* have been reported with advancing liver dysfunction and cirrhosis [40]. In rodent studies, cecal microbiota from ob/ob mice when introduced into germ free wild mice resulted in modest fat gain and increased food calorie extraction when compared to mice receiving gut microbiota from lean donors [41]. Methods to exogenously modify gut microbiota have also been tested with encouraging results. When adult patient with biopsy proven hepatic dysfunction were treated with a combination of orally administered Lactobacillus bulgaricus and Streptococcus thermophilus for three months, there was a significant decrease in ALT, AST and gamma glutamyl transferase (GGT) levels compared to controls [42].

Animal studies have demonstrated an increase in Bacteroidetes compared to Firmicutes in TPN infused animals and that colonization with Bacteroidetes leads to intestinal inflammation [43,44]. Such bacterial colonization can also result in increased intestinal permeability [45,46]. Increased bacterial infiltration across the gut causes endotoxin and cytokine mediated down regulation of bile acid transporters and ultimately hepatic injury [47,48,49]. Significantly higher Tumor Necrosis Factor (TNF) and Interleukin-6 (IL-6) levels have been noted in animals on TPN [50,51,52]. Multiple investigators have reported a decrease in inflammation induced liver injury in rats upon initiation of oral antibiotics, suggesting a role of bacteria in the development or exacerbation of PNALD [53,54,55]. 

## 5. FXR and TGR5 Regulated Gut Integrity

Emerging data points to a gut protective role of FXR agonists. Mice lacking FXR have been shown to have increased ileal levels of bacteria and a compromised epithelial barrier and it has been implicated that FXR agonists may prevent epithelial deterioration and bacterial translocation [56,57].

Recent data from experimental colitis, implicates modulation of the intestinal barrier and immune responses by TGR5, postulating the noted effects being secondary to a change in the gut microbiota [58].

One of the most notable FXR modulated gene is inducible nitric oxide synthase (iNOS) [56,59]. Other FXR regulated genes include those coding for angiogenin (ANG1), a part of the acute phase response to infection, which has potent antibacterial actions. Carbonic anhydrase 12 (CAR12), involved in antibacterial defense by regulating luminal pH and ion balance is also modulated by FXR [60,61]. TGR5 activation is known to lower pro-inflammatory cytokines interleukin-1α (IL-1α), IL-1β, IL-6 and tumor necrosis factor-α (TNF-α) [62].

Therefore it remains plausible that a dysbiotic clonal expansion during TPN therapy causes increased gut permeability and hence endotoxin, cytokine mediated injury, which could be potentially prevented by FXR and TGR5 agonists.

## 6. Role of the Lipid Emulsion

The exact mechanisms of lipid induced liver injury remain poorly understood. Due to their rich content of essential fatty acids, traditionally, vegetable oils have been used as a source of lipids during parenteral nutrition. The primary source for lipids thus has been soybean oil derivatives [63]. As opposed to the soybean derived emulsions, with a predominance of ω-6fatty acids, fish oil based emulsions are higher is ω-3fatty acids [64]. Several studies have shown beneficial metabolic effects of ω-3fatty acid based lipid solutions in preventing or attenuating hepatic steatosis and cholestasis [65,66]. A study in neonatal piglets compared hepatic outcomes when using parenteral nutrition containing a 100% soy based emulsion (ω-6:ω-3 PUFA: 7:1) or a mixed lipid emulsion containing soy oil, medium-chain triglycerides (MCTs), olive oil, and fish oil (ω-6:ω-3 PUFA: approximately 2.5:1). Prevention of liver disease and a reduction in systemic inflammation was noted with the higher ω-3 based formulation [67].

In another study using neonatal piglets assessing if fish oil based lipid formulations could prevent PNALD, animals were given parenteral nutrition containing 100% soybean oil, 100% fish oil or a lipid mixture (soy oil, MCTs, olive and fish oil). After 14 days of treatment plasma levels of direct bilirubin, GGT and bile acids were significantly lower in animals receiving fish oil based emulsions with a greater reduction in direct bilirubin in animals on 100% fish oil [68].

Data from human studies using fish oil based emulsions have also been encouraging. Significant reduction in mortality and the need for liver transplantation has been noted in pediatric patients receiving fish oil based emulsions in comparison to those given plant derived emulsions [69,70].

While there have been concerns for essential fatty acid deficiency with 100% fish oil based emulsions, recent studies have shown otherwise [71,72]. There is emerging data that indicates that fish oil based monotherapy results in platelet dysfunction [73], which may be of clinical relevance in the context of human TPN administration with such emulsions. Additionally, a recent publication suggests that improvement noted with a commercial ω-3 based emulsion may be due to significantly higher levels of Vitamin E, however further studies elucidating mechanistic links may be needed [74].

High ω-6fatty acid based emulsions are known to impact immune function by increasing cytokine production by activating the nuclear factor-kB pathway, which disrupts hepatobiliary transport leading to cholestasis [75]. Additionally high ω-6fatty acids accentuate lipid perioxidation and deplete levels of anti-oxidant tocopherols [70,76]. Prospective studies have noted improvement in systemic inflammation, as well as improvement in parenteral nutrition associated liver disease with use of fish oil based lipids [67,77].

There is also emerging evidence of the inhibitory effects of phytosterols found in soy-based lipids on bile acid secretion and excretion. Preterm infants on soy based lipid emulsions have significantly higher levels of phytosterols compared to controls and poorly developed mechanisms eliminating phytosterols have been implicated in their vulnerability to PNALD [78]. It has been postulated that phytosterols augment hepatic inflammation through TLR4 (Toll Like Recptor 4) macrophage activation. Addition of stigmasterol (a phytosterol) to PN solutions has been associated with proinflammatory hepatic macrophage activation [79,80]. Phytosterols also exert an inhibitory effect on FXR [81,82,83,84] In fact stigmasterol antagonizes the bile acid activated FXR target genes Bile salt export pump (BSEP) and the orphan nuclear receptor Short heterodimer partner (SHP) in FXR +/+ mice but fails to do so in FXR −/− mice hepatocytes [82]. The disrupted FXR signaling secondary to phytosterols has been additionally implicated in the metabolic dysregulation of bile acid pathways contributing to hepatic injury.

There have also been studies evaluating the role of the amount of the lipid provided as a contributor to PNALD. Though traditionally lipids have been provided at 2–3 grams/kg/day, there have been studies demonstrating a reduction in the incidence of PNALD with a lipid dosed at ≤1 gram/kg/day [85,86]. Though this strategy has merits, there have been concerns for deleterious effects due to a lack of lipids in the growing infant. A higher Triene:Tetraene ratio has been noted in infants on lipid restricted parenteral nutrition indicating a trend towards a deficiency of essential fatty acids [85,87,88].

## 7. Toxicity of TPN Solution

Data suggests that toxicity as well as relative deficiencies of parenteral nutrition components can lead to TPN associated injury. Aluminum, chromium and manganese have all been implicated. Aluminum present in the TPN solution is known to cause metabolic bone disease as well as neurological impairment [89]. In infant studies plasma concentration of aluminum has been shown to be several fold higher in patients receiving TPN *vs.* those on enteral nutrition [90].

Concerns have also been raised for organ damage secondary to chromium delivery during parenteral nutrition. Chromium plays a role in regulation of the action of insulin. Additionally, peripheral neuropathy, weight loss as well as kidney damage have been reported in patients receiving TPN. Over the past several years there has been an effort to decrease chromium concentration in parenteral nutrition solutions [91].

Anemia, cholestasis as well as neurotoxicity have been noted with manganese provided as part of TPN. Recent guidelines recommend monitoring of manganese levels if TPN has been provided for longer than 30 days. However, controversy exists in the method for assessing manganese stores as a biomarker of manganese associated toxicity. Whole blood levels of manganese are highly variable and do not stringently correlate with manganese toxicity [92]. Reduction in manganese levels in TPN solution has been shown to reduce intra organ manganese deposition and thus may help in preventing toxicity [93].

Diets with carbohydrate excess have also been noted to enhance liver injury during parenteral nutrition [94]. Even though dextrose is not considered directly hepatotoxic, it has been speculated that there is enhanced insulin release and a resulting upregulation of enzymes regulating fatty acid synthesis with excessive carbohydrates. This causes hepatic steatosis leading to inflammatory hepatic damage [95,96,97].

## 8. Prematurity

Clinical evidence points to significantly higher incidence of TPN associated pathology in premature babies in comparison to older individuals [98]. The mechanistic basis for such differences are not clearly delineated, however, it is postulated that immaturity in bile acid transporters regulating entero-hepatic circulation may be key contributors [99]. The expression of multidrug resistance protein 3, which is involved in phospholipid excretion in bile, was significantly less in human fetal livers in comparison to adult livers. The study also found significantly reduced levels of the Sodium/bile acid co-transporter protein, which is a key glycoprotein involved in normal entero-hepatic circulation [100,101,102]. Additionally, the expression of BSEP, which modulates the rate-limiting step of bile salt transport driving the enterohepatic circulation, though noted in early gestation, gradually increases with gestation age [101,102].

## 9. Conclusions

TPN infusion is associated with significant morbidity and mortality. Though several postulated mechanism have been noted and extensively researched, there appears to be a broad support for the hypothesis that TPN associated pathology results from an alteration of the normal enterohepatic circulation. Several other mechanisms as detailed in this review are likely contributors to varying degrees.
